# Novel Aspects of the Liver Microenvironment in Hepatocellular Carcinoma Pathogenesis and Development

**DOI:** 10.3390/ijms15069422

**Published:** 2014-05-27

**Authors:** Thomas Tu, Magdalena A. Budzinska, Annette E. Maczurek, Robert Cheng, Anna Di Bartolomeo, Fiona J. Warner, Geoffrey W. McCaughan, Susan V. McLennan, Nicholas A. Shackel

**Affiliations:** 1 Liver Cell Biology, Centenary Institute, Sydney, NSW 2050, Australia; E-Mails: t.tu@centenary.org.au (T.T.); m.budzinska@centenary.org.au (M.A.B.); a.maczurek@centenary.org.au (A.E.M.); r.cheng@centenary.org.au (R.C.); f.warner@centenary.org.au (F.J.W.); g.mccaughan@centenary.usyd.edu.au (G.W.M.); 2 A.W. Morrow Gastroenterology and Liver Centre, Royal Prince Alfred Hospital, Sydney, NSW 2050, Australia; 3 Sydney Medical School, University of Sydney, Sydney, NSW 2006, Australia; E-Mail: sue.mclennan@sydney.edu.au; 4 School of Medicine, University of Adelaide, Adelaide, SA 5005, Australia; E-Mail: anna.dibart@gmail.com; 5 Department of Endocrinology, Royal Prince Alfred Hospital, Sydney, NSW 2050, Australia

**Keywords:** cancer, liver, microenvironment, hepatocellular carcinoma

## Abstract

Hepatocellular carcinoma (HCC) is a prevalent primary liver cancer that is derived from hepatocytes and is characterised by high mortality rate and poor prognosis. While HCC is driven by cumulative changes in the hepatocyte genome, it is increasingly recognised that the liver microenvironment plays a pivotal role in HCC propensity, progression and treatment response. The microenvironmental stimuli that have been recognised as being involved in HCC pathogenesis are diverse and include intrahepatic cell subpopulations, such as immune and stellate cells, pathogens, such as hepatitis viruses, and non-cellular factors, such as abnormal extracellular matrix (ECM) and tissue hypoxia. Recently, a number of novel environmental influences have been shown to have an equally dramatic, but previously unrecognized, role in HCC progression. Novel aspects, including diet, gastrointestinal tract (GIT) microflora and circulating microvesicles, are now being recognized as increasingly important in HCC pathogenesis. This review will outline aspects of the HCC microenvironment, including the potential role of GIT microflora and microvesicles, in providing new insights into tumourigenesis and identifying potential novel targets in the treatment of HCC.

## 1. Introduction

Liver cancer is the fifth most common malignancy globally [1,2,3]. Primary liver cancers include cholangiocarcinomas, hepatoblastomas, and hepatocellular carcinomas (HCC), of which the latter accounts for >90% of primary liver cancers with 500,000–1,000,000 new cases being diagnosed globally annually [1,4,5,6]. HCC is associated with a poor five-year survival rate, leading to ~600,000 deaths each year [7,8]. The worldwide distribution of HCC patients correlates strongly with the incidence of chronic hepatitis B virus (HBV) and hepatitis C virus (HCV) infections, which are present in 75%–85% of people with HCC [5,9,10,11]. The four major risk factors for HCC include: (1) underlying cirrhosis; (2) chronic HBV infection; (3) chronic HCV infection; and (4) exposure to the food-borne mycotoxin aflatoxin B1 (AFB1) [5,12,13,14]. HCC generally occurs late in life and on a background of progressive chronic liver injury, typically over years to decades [3]. In the past, it was generally accepted that HCC occurs as a result of the transformation of hepatocytes, the main cell of the liver. However, the multitude of subtypes and phenotypes of HCC, e.g., progenitor cell phenotype [15], suggest that HCC can originate from other liver cell types including liver progenitor cells [16]. In this review, we will discuss the growing body of evidence that the progression of HCC is not only due to the molecular changes in hepatocytes, but is also influenced by an increasing list of mediators in the liver microenvironment, *i.e.*, surrounding non-hepatocyte cells, non-host microbes (e.g., the GIT microflora) and non-cellular factors (e.g., microvesicles).

## 2. Overview

This review is divided into four main sections:

1. A discussion of the molecular alterations that occur with HCC.
2. A review of the well-established microenvironmental contributors to HCC and their mechanisms.
3. A description of novel microenvironmental factors whose effects on HCC initiation and development are incompletely understood.
4. An exploration of the clinical impact of novel microenvironmental contributors to HCC initiation and development.

## 3. Hepatocellular Carcinoma

### 3.1. Background

**Figure 1:**
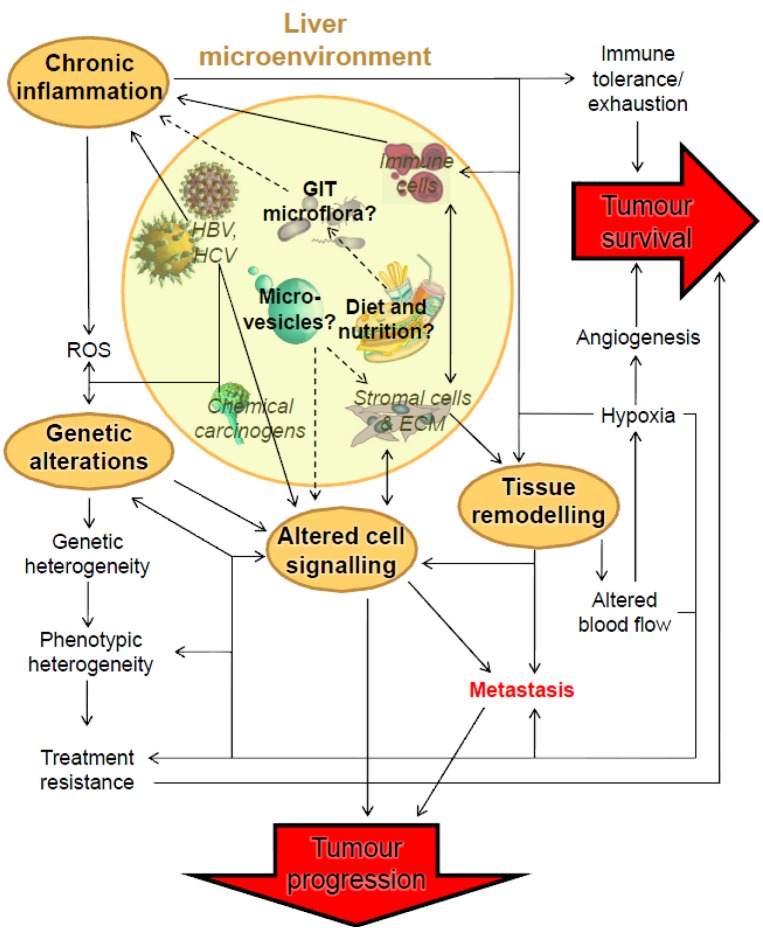
A simplified network of liver microenvironmental factors and their roles in HCC tumour progression and maintenance. Established causal interactions between microenvironmental factors (italicised within the dashed circle) and HCC tumour progression and maintenance are represented by solid arrows, while dashed arrows are interactions that have not yet been conclusively shown. Some known interactions are not shown for clarity. In this review, we describe the direct effects of the liver microenvironment on this network (bold): chronic inflammation; tissue remodeling; genetic alterations; and altered cellular expression. ROS = Reactive Oxygen Species; HBV = Hepatitis B Virus; HCV = Hepatitis C virus; GIT = Gastrointestinal Tract; ECM = Extracellular Matrix.

The composition of the liver includes hepatocytes and non-hepatocellular cell types [17,18]. Hepatocytes are the main functional parenchymal liver cells that perform the majority of the metabolic duties of the liver. The non-hepatocyte cell types include: (1) stromal cells, such as hepatic stellate cells and fibroblasts, which play a structural role and are responsible for ECM production; (2) immune cells, which mainly consist of resident macrophages (Kupffer cells) in physiological settings, but can include a range of inflammation-recruited leukocytes; (3) epithelial cells lining the bile ducts (cholangiocytes); (4) endothelial cells, which line the blood vessels (liver sinusoidal endothelial cells; LSECs); and (5) liver progenitor cell populations (called oval cells in mice) which give rise to both hepatocyte and non-hepatocyte intrahepatic cell populations. The non-hepatocyte cell types may influence both the initiation and progression of HCC [16,19]. Infectious pathogens, including hepatitis viruses, also contribute to malignant intrahepatic transformation [9,12,20]. Further, non-cellular factors, which include hypoxia and altered ECM, can drive the transformation process through paracrine signaling by surrounding stromal tissue [21]. The contribution of all of these established factors to the development and maintenance of malignant cells is highly interconnected (Figure 1) and has been described in detail in several reviews [22,23,24].

### 3.2. HCC Diagnosis and Treatment

HCC is diagnosed by a combination of ultrasound imaging, computer tomography (CT) imaging, magnetic resonance imaging (MRI) and measurement of serum alpha-fetoprotein (AFP) levels [25]. Implementation of surveillance programs in high-risk patients has led to increased diagnosis of HCC in early (defined by the Milan criteria as a solitary tumour <5 cm, or <3 tumours, with all tumours <3 cm), asymptomatic stages [26]. If HCC is detected in this stage, surgical interventions—*i.e.*, partial liver resection or liver transplant—are available as effective curative treatments for HCC [27]. In these cases, the median 5-year survival rate is ~70% [28,29].

Despite these programs, diagnosis of HCC still usually occurs in the late stages of progression [10,11,30], a factor which likely contributes to the poor (20%–25%) response rate to treatments with curative intent [10,11,30,31]. Treatments at late stages to control HCC burden or progression—such as administration of the small molecule tyrosine-kinase inhibitor Sorafenib, percutaneous ethanol injection, transarterial chemoembolisation (TACE), and radiofrequency ablation—are rarely curative [32,33]. The chemoresistance associated with HCC is caused by multiple mechanisms: the up-regulation of pathways in the tumour to limit uptake or induce metabolism of chemotherapeutics; the inhibition of chemotherapeutic activity due to the hypoxic environment of the tumour and cirrhotic tissues; and potentially the slow-dividing “HCC stem cell” niche that is intrinsically chemoresistant towards drugs targeted at tumour cells [34,35,36,37]. The main causes of death in these HCC patients are tumour progression, upper gastrointestinal bleeding or hepatic failure [38,39].

Even when HCC is detected in early stages, post-treatment recurrence is common and occurs in ~15% of patients treated with liver transplant [40,41,42], and ~60% of patients treated with partial resection with curative intent [43,44,45,46]. The median 5-year survival rate after recurrence is ~20%–35% [47,48,49,50]. Two types of recurrence have been reported: early recurrence, which occurs within 2 years of treatment and is assumed to arise from tumour cells not removed by liver resection or other treatments; and late recurrence, which generally occurs in a distant site from the primary tumour and is assumed to originate from an independent population of transformed hepatocytes [51,52]. Intrahepatic recurrence—as opposed to metastasis to extrahepatic tissues—occurs in the majority of cases, suggesting that the pro-oncogenic changes in the microenvironment of the resected liver play a major role in recurrence [53,54,55]. This hypothesis is supported by several reports showing that gene-signatures, including those centred about altered HCC-associated interferon (IFN)-γ and p53 signaling networks, in non-tumour tissue can be measured as an indicator for the probability of recurrence [56,57,58].

### 3.3. Histological and Molecular Changes

HCC has a highly variable histological appearance [59]. Identification of tumour cells is based on the presence of more than one of the following changes: high nuclear to cytoplasmic ratio; prominent nuclei and nucleoli; nuclear irregularity; moderate eosinophilia; and finely granular cytoplasm [60]. Additionally, normal, single cell-thick sinusoid structures are replaced with ≥2 cell-thick contorted cords. As HCC progresses, apparent dedifferentiation of the cells occurs from a hepatocyte-like morphology to a more stem-cell like phenotype [61,62]. For this reason, progression of HCC is associated with histological dedifferentiation from well-, moderately- to poorly-differentiated morphology. The extent of dedifferentiation of hepatocytes assessed by morphology correlates with poorer patient survival and increased rate of tumour recurrence [63,64,65]. HCC progression has also been associated with changes in subcellular organelles, such as significantly fewer and smaller mitochondria, and greater amounts of endoplasmic reticulum and free ribosomes [66,67,68]. Further, mitochondrial DNA mutations (though not consistently in any particular gene) have been detected in HCC [68,69,70]. These organelle changes may be due to metabolic alterations—such as increased autophagy or the Warburg effect—associated with carcinogenesis, but this has yet to be shown.

On a molecular level, HCC genomes commonly have alterations in the DNA genome, including DNA mutations in multiple oncogenes and tumour suppressor genes [71,72,73,74,75,76] and changes in chromosomal copy number, e.g., loss of 1q21.2 and gain of 17q13.1 [77,78], even in varying aetiologies [79,80,81]. Epigenetically, CpG methylation has been seen in the gene promoters of the JAK/STAT and other HCC-driving pathways, though these changes have also been seen in non-tumour tissue [82,83,84]. Additionally, alterations in histone-altering enzymes, such as histone deacetylases and histone methyltransferases, have been implicated in altering expression in HCC pathways though the mechanisms by which they achieve this is not entirely understood [85,86,87,88]. In this section, the most common and well-described changes associated with HCC are described briefly, though it should be noted that this list of altered pathways is not complete, and that these carcinogenic pathways are complex, interconnected, and, in some cases, still to be completely characterised. More detailed descriptions of these pathways are beyond the scope of this review and have been well summarised elsewhere (see relevant cited references).

One of the canonical molecular changes associated with HCC is the differential expression of the Wnt pathway, which regulates the expression of many proliferation, metabolism, and ECM remodeling-associated genes, such as c-myc, c-jun, glycogen synthase kinase-3β, cyclin D1 and matrix metalloproteinases (MMP) [72,89,90,91]. The Wnt pathway has been reported to be up-regulated in up to 90% of HCC tumours [75,76]. Wnt pathway genes may act in concert with the pathway associated with tumour suppressor gene p53, which has been observed to be commonly mutated in primary HCC tissue and animal injury models [92]. However, alteration of the Wnt or p53 pathway is not sufficient for HCC development, as the activation of these pathways via altered p53, adenomatous polyposis coli, or β-catenin gene expression has also been observed in benign liver neoplasms [93] and non-tumour tissue surrounding HCC tumours [94,95]. Additionally, alterations in the Wnt signaling pathway are significantly less common in HBV-associated HCC compared to other aetiologies, suggesting that further changes in the cell phenotype are necessary to produce HBV-associated HCC [76,96].

Another pathway altered in HCC is the Hedgehog (Hh) signaling pathway, which is up-regulated in up to 60% of HCC tumours [97]. Under normal conditions, the Hh pathway is involved in embryonic tissue development through regulation of a large range of downstream target genes [98]. In cancer, inhibition of the Hh pathway has been shown to decrease proliferation and increase apoptosis in HCC cell lines where Hh is regularly up-regulated [97,99,100]. Furthermore, studies from our group have shown a significant increase in intrahepatic levels of Hh mRNA in liver cirrhosis of different aetiologies compared to healthy control livers [101,102]. Similar to the Wnt signaling pathway, the Hh signaling pathway has been found to be up-regulated in a fraction of non-tumour tissue and is not seen in all HCC tumours [97], suggesting that it is also neither sufficient nor necessary for liver carcinogenesis.

Similar changes are seen in the JAK/STAT pathway with the activating phosphorylation of the key transcription factor STAT3 being reported in 50%–100% of HCC tumours and rarely in surrounding non-tumour tissue [103,104,105]. Activation of this pathway is associated with multiple cell functions, such as differentiation, proliferation, and apoptosis [106]. DNA mutations in genes of this pathway are rare, suggesting other regulatory mechanisms are involved, such as DNA promoter methylation or other epigenetic mechanisms [105]. The JAK/STAT pathway can also be activated by excess cytokine signaling via, for example, IL-6 [107,108], which reinforces the point that the liver microenvironment can directly stimulate the development of HCC and should be considered in HCC progression.

While there does not appear to be any single pathway that is sufficient or necessary for HCC development, these pathways commonly result in up-regulation of genes involved in cell cycle progression, proliferation and escape from apoptosis [109,110,111,112]. Not only does it allow for faster tumour growth and escape from some DNA proofreading mechanisms [113], overcoming cell cycle checkpoints is particularly important for HCC progression in the liver microenvironment where hepatocyte turnover is tightly regulated [114,115].

**Table 1:** Previously reported HCC subtypes and their associated clinical outcomes.

Subtype	Clinical outcome associated with subtype	References
Hepatoblastoma-like	Shorter time to post-resection HCC recurrence	[19]
	Poorer overall survival	
Cholangiocarcinoma-like	Shorter time to post-resection HCC recurrence	[116]
	Poorer overall survival	
Cytokeratin-19 (CK19)-positive	Shorter time to post-resection HCC recurrence	[117]
	Poorer overall survival	
AFP-positive	Increased HCC invasion	[118,119]
	Poorer overall survival	
Notch-positive	No correlation to clinical outcome	[120]
Retinoblastoma-positive	Better overall survival	[121,122]
CD24-positive	Shorter time to post-resection HCC recurrence	[123]
	Poorer overall survival	
Transforming Growth Factor (TGF)-β late signature (compared to early signature)	Increased HCC invasion	[124]
	Increased risk of HCC metastasis	
	Poorer overall survival	
Myc TGF-α-positive	Poorer overall survival	[125]
Chromosome 7 gain	Shorter time to post-resection HCC recurrence	[119]
Subtypes A and B based on chromosomal copy number alteration	Subtype A is associated with poorer overall survival	[126]

Despite this, it has been difficult to explain much of the heterogeneity observed in HCC tumours. Transcriptomic analysis has shown that changes in these common pathways occur late in the tumour development, while only modest transcriptomic changes are seen in early HCC and dysplastic nodules [127]. Even in late stage, intra-tumoural heterogeneity is observed in HCCs, leading to the classification of distinct HCC subtypes (Table 1). However, there is considerable variation even within each of these subtypes. It has been suggested that some of these subtypes arise from a difference in the cell of origin of a particular HCC. In particular, hepatoblastoma-like and cytokeratin-19 (CK-19) positive tumours are hypothesised to have originated from liver progenitor cells due to the similarity of their transcriptomic signatures [19,128,129,130,131]. However, novel data shows that the cells from which HCC originates do not necessarily determine the final phenotype of the tumour [16]. Thus, the cell of origin in HCC, whether from hepatic progenitor cells, dedifferentiated hepatocyte, extra-hepatic haematopoietic stem cells or a combination of some or all of these, is currently controversial. Due to the difficulty in determining the cell of origin, especially in primary clinical samples, this research is often hampered and so this controversy is not likely to be quelled soon. 

In response to the heterogeneity displayed between and within the various HCC subtypes, large panels of genes have been used to define risk for HCC initiation, progression and recurrence, instead of single genes or pathways. For example, an mRNA expression panel of 186 genes has been found to be predictive of both HCC recurrence post-transplantation and of HCC initiation in HCV-infected patients with early cirrhosis [58,132], independent of other clinical features. One of the most successful panels for HCC progression includes a 5-gene panel, based on combined expression level of the genes HN1, RAN, RAMP3, KRT19 and TAF9, that has been shown to be highly predictive of survival time post-resection in HCC patients in multiple cohorts [133].

## 4. Microenvironmental Influence on HCC Pathogenesis

Research examining HCC has focussed on the histological and molecular changes associated with tumour propensity or behaviour. However, it is clear that the microenvironment within the liver should also be considered for both HCC initiation and progression. With regard to initiation, numerous studies and reviews have suggested that circulating cancer cells do not simply grow wherever they are seeded in the body, but their fate (e.g., quiescence or metastatic growth) is determined by the microenvironment of the seeded tissue, often referred to as the “seed and soil” hypothesis [134,135,136]. Additionally, the existence of the different subtypes of HCC [137] suggests that the initiation and progression of HCC may be shaped by various selective pressures, defined by the liver microenvironment.

In the following sections, we will review the roles of the various aspects of the liver microenvironment reported to drive HCC progression and tumour survival (Table 2). As shown schematically in Figure 1, the HCC tumour microenvironment is a highly-interconnected network. In this review, we will focus on particular nodes driven by the liver microenvironment: chronic inflammation, tissue remodeling, genetic alterations, and alterations in cellular signaling.

**Table 2:** Established and novel elements of the liver microenvironment and their roles in driving HCC progression [138,139,140,141,142,143,144,145,146,147,148,149,150,151,152,153,154,155,156,157,158,159,160,161,162,163,164].

Microenvironmental element	Mechanisms	Example references
Immune cells	Activation of inflammatory cells leading to hepatocyte turnover, ROS formation	[138]
	Inhibition of immunosurveillance	[139]
	Regulatory T-cell (T_reg_) and tumour-associated macrophage (TAM) inhibition of cell-mediated cytotoxicity and immune responses against tumour cells	[140,141,142]
Chemical carcinogens (e.g., Aflatoxin B1)	DNA adduct formation, leading to chromosomal instability	[143,144,145]
	Direct induction of cell cycle entry	[146]
Hepatitis B/C viruses	Induction of chronic inflammation	[147]
	Induction of immune tolerance	[148,149]
	Formation of reactive oxygen/nitrogen species	[150,151,152]
	Alteration of DNA repair mechanisms via viral proteins	[153]
	Direct alteration of cellular pathways by viral proteins	[154,155,156,157,158,159]
Hypoxia	Alteration of macrophages to TAM-like phenotype, leading to immunosuppression and angiogenesis	[141,160,161]
	Induction of angiogenic cytokines by hepatocytes/tumour cells	[162,163]
Altered stromal cells and ECM changes	Build-up of ECM (via altered ECM composition) leading to hypoxia	[164]
	Release of pro-tumourigenic cytokine TGF-β by altered stromal cells	[21]
	Impeding access of chemotherapies to tumour	[34]
Diet and nutrition	Unknown	
GIT microflora	Increasing permeability of gut to pro-inflammatory and pro-oncogenic bacterial metabolites (e.g., lipopolysaccharide and deoxycholic acid) responses	[165,166]
Extracellular microvesicles	Carry ECM-altering signals	[167,168]
	Carry oncogenic miRNAs and cytokines	[169,170,171,172]
	Induction of immunosuppression	[173,174]

### 4.1. DNA Alterations in HCC

Several microenvironmental factors can directly induce the DNA alterations that are associated with the stepwise progression of HCC [143,144,145]. Chemical exposure and dietary ingestion of carcinogens are known to directly alter DNA sequences and cause DNA mutation [175,176,177]. For example, the chemical carcinogen 2-acetylaminofluorene (2-AAF) causes DNA adducts in mice [175]. AFB1, a mycotoxin present in a wide range of foods, is also a direct carcinogen that causes DNA adducts and mutations via DNA base conversion from G to T [177]. In particular, human exposure to AFB1 has been epidemiologically associated with mutations in the tumour suppressor gene p53 in HCC patients [176]. Further, mutations in p53 have been recapitulated in transgenic mouse fibroblasts by *in vitro* exposure to AFB1 [178]. This AFB1-associated DNA alteration can also become a feed-forward mechanism as AFB1 suppresses G1 checkpoint arrest by p53, bypassing the cellular DNA damage response, which leads to a greater mutation rate [179]. Additionally, the combination of AFB1 exposure and p53 mutation has been associated with chromosomal instability with HCC, further contributing to DNA alterations [180]. Further, the genetic diversity introduced by increased DNA mutations is postulated to decrease the effectiveness of chemotherapeutic agents, by increasing the likelihood that resistant tumour cells exist prior to chemotherapy [181,182]. Studies have also suggested that HCV gene products may directly interfere with cellular DNA repair mechanisms to induce DNA mutations [153].

### 4.2. Chronic Inflammation

Chronic inflammation is one of the main contributors to HCC initiation and progression. In virtually every case, chronic inflammation precedes the development of HCC [138]. The microenvironmental causes of chronic inflammation are diverse, including chronic HBV and HCV infection [147], and excessive ethanol consumption [147,183,184]. The immune-mediated cell death associated with chronic HBV and HCV infections can continuously trigger production of ROS, including hydrogen peroxide, hydroxyl radicals and superoxide radicals, by macrophages, neutrophils and cytotoxic T-lymphocytes [147]. The increased ROS results in increased hepatocellular oxidative stress, which can in turn induce DNA mutations that drive HCC [185,186,187,188]. HCV can directly increase intracellular ROS via expression of the HCV core protein and its inhibition of mitochondrial electron transport [148,149]. Ethanol consumption, a risk factor of HCC, is also known to increase the ROS concentration within hepatocytes, leading to increased formation of DNA adducts [147,183,184]. Additionally, reactive nitrogen species, such as nitrous oxide, are produced by hypoxic hepatocytes, the oxidative burst of infiltrating immune cells and endothelial cells responding to altered blood flow within the tumour or cirrhotic tissues [189].

These microenvironmental sources of chronic inflammation cause increased proliferation of hepatocytes, which leads to an increase in DNA mutations due to susceptibility for DNA damage during mitosis [190,191]. The subsequent proliferation of hepatocytes leads to a shortening of telomeres, which is in turn associated with chromosomal instability and progression to HCC [192]. The carcinogen diethylnitrosamine (DEN) induces proliferation of hepatocytes by up-regulating G1/S-phase regulatory proteins in mice [146] and can lead to HCC when given along with 2-AAF [193]. Another characteristic of chronic inflammation is tissue remodeling, which manifests as fibrosis and ultimately cirrhosis. In particular, chronic inflammation can alter cytokine expression within the injured liver, leading to an excess of ECM synthesis by hepatic stellate cells and resultant ECM accumulation [194,195,196]. The significance of tissue remodeling in the progression of HCC is described in further detail below.

Severe chronic inflammation can lead to the expansion of liver progenitor cell populations in the liver to replace hepatocytes [197]. As these are hypothesised to be the origin of at least a fraction of HCCs, proliferation of liver progenitor cells may increase the risk of HCC. Further, the cytokine milieu associated with chronic inflammation (e.g., elevated IL-6 levels) may drive liver progenitor cells to a more cancerous phenotype [198].

Chronic inflammation can also lead to tolerance or exhaustion of the cell-mediated immunity [139] which contributes towards the development and progression of HCC. The most obvious example of the importance of immune surveillance in HCC development is seen in liver transplantation where immunosuppression is used to prevent rejection. Post-transplant HCC recurrence is aggressive and early reduction in immunosuppression is used to minimize recurrence likelihood and/or severity [199,200]. Indeed, calcineurin inhibition of T-cell function to reduce organ rejection is thought to be one of the principal causes of the aggressiveness of HCC recurrence in the post-transplant setting [199,201]. Therefore, lymphocytes, in particular T-cells, are critically important in the immune surveillance and suppression of HCC. Tumour evasion of the immune response can also be facilitated by the intrinsic tolerogenic microenvironment of the liver [202,203], caused in part by Kupffer cells, the main population of macrophages resident in the liver [204,205,206]. Chronic virus infections, such as HBV and HCV, can also contribute to this tolerogenic effect and repression of cytotoxic T-cell response through secretion of viral antigens interacting with immune cells and altering signaling between the host cell and immune system [150,151,152]. Current evidence also suggests that HCC tumour cells can directly alter the liver microenvironment by recruiting regulatory T-cells (T_reg_) [207,208], or up-regulation of the programmed death-1 (PD-1) pathway [209,210,211], which have been associated with both poorer patient outcome and increased post-operative recurrence [208,212,213].

### 4.3. Tissue Remodeling and Stromal Interactions

**Figure 2:**
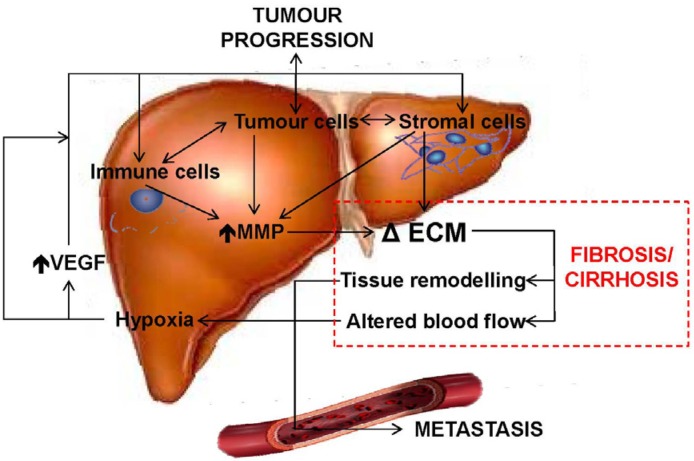
A schematic diagram of interactions between the ECM and cells in the liver microenvironment.

Remodeling of the ECM in HCC involves the concerted action of multiple cell types in the liver microenvironment, including: (1) stromal cells, such as fibroblasts and hepatic stellate cells, which can lay down ECM components; (2) immune cells, such as macrophages, which can induce the ECM remodeling through activation of stromal cells or directly degrade ECM via the actions of MMPs; and (3) tumour cells, which can directly induce remodeling by stromal cells through altered signaling molecules/receptors (Figure 2). These microenvironmental factors can stimulate increased ECM accumulation by changing the balance between ECM degradation and ECM synthesis. As mentioned previously, chronic inflammation can stimulate hepatic stellate cell ECM synthesis and resultant ECM accumulation [194,195,196]. Also, the switch from hepatic stellate cell synthesis of collagen-IV in normal liver to the more proteolysis-resistant collagen types-I and -III also plays a role by slowing down ECM degradation [214,215]. Further, increased expression of tissue inhibitors of MMPs (TIMPs) is associated with ECM build-up [216]. Excess ECM is classified as fibrosis, and then progresses to cirrhosis once the excess fibrotic bands begin to enclose entire liver lobules. As cirrhosis is present in the majority (80%–90%) of patients with HCC [14], the characteristic excess of fibrotic ECM in cirrhosis suggests that abnormal ECM accumulation is also a key driver of HCC progression, though no direct causal link has been convincingly demonstrated.

Altered blood flow and resultant hypoxia that occurs due to this ECM accumulation are stimulators of tumour progression. For example, tumour growth occurs rapidly following hepatocyte transformation, but is soon limited by the diffusion of oxygen and nutrients when the tumour reaches ~1 mm^3^ [217]. Tumour and non-tumour cells respond to hypoxic conditions by up-regulating angiogenic factors, such as vascular endothelial growth factor (VEGF) [162,163]. Hepatic stellate cells and liver endothelial cells respond to VEGF by stimulating vascular growth, which alleviates the limited supply of oxygen and nutrients to the tumour and thereby promotes tumour growth. Overexpression of VEGF by both tumour and surrounding non-tumour cells are responsible for the chaotic angiogenesis in tumours [218,219]. This uncoordinated angiogenesis is generally insufficient to relieve tumour-associated hypoxia, so areas of hypoxia still exist and this, in turn, leads to even greater angiogenesis. Altered hepatic blood flow can also reduce the amount of active therapeutic agents that can reach the HCC tumour, thus increasing its resistance to chemotherapy [34].

The pockets of hypoxia in the liver parenchyma during cirrhosis have also been proposed to be a driving mechanism of HCC [164]. Areas of hypoxia can lead to changes in molecular signaling in non-tumour cells that induce the progression of HCC. Macrophages are known to accumulate in hypoxic regions and their activation in hypoxic conditions induces a tumour-associated macrophage (TAM) phenotype [141,160,161]. This phenotype is associated with increased expression of tolerogenic molecules, such as PD-1 ligand and interleukin-10, and pro-angiogenic factors, such as angiopoietin 1, VEGF, and MMP-9 [140,141,142]. However, current reports conflict on whether increased intra-tumoural TAMs are associated with a favourable [220] or unfavourable [221] outcome in HCC patients.

### 4.4. Altered Hepatocyte Cell Signaling

Altered hepatocyte-stromal cell crosstalk in progressive fibrosis, consisting of alterations in tumour phenotype by ECM components or *vice-versa*, is also important in HCC pathogenesis [222,223]. For example, altered stromal cells have been reported to drive HCC development via direct signaling to the hepatocytes. Transfer of inflammation-induced myofibroblasts, *i.e.*, fibrogenic cells derived from hepatic stellate cells, was shown to promote HCC in tumour suppressor gene *p19^ARF^*-knockout mice [21]. This promotion was suggested to occur through TGF-β secretion from the implanted stromal cells activating oncogenic pathways in the tumour cells.

Tumour-initiated alterations in ECM interactions have been associated with HCC progression and metastasis. Known pathways include down-regulation of cadherins (specifically E-cadherin), up-regulation of MMP activity and epithelial–mesenchymal transition (EMT) [224,225,226]. Alterations in all of these pathways are at least partly mediated by the expression of Extracellular Matrix Metalloproteinase Inducer EMMPRIN. EMMPRIN (also known as Tumour-derived Collagenase Stimulatory Factor or CD147) is a glycoprotein involved in MMP regulation at the tumour-stromal interface [227]. EMMPRIN expressed by leukocytes, tumour cells and TAMs induces contacting hepatic stellate cells [228,229] and hepatocytes [230] to increase expression of MMPs. Previous studies from our group have shown that intrahepatic EMMPRIN mRNA is increased in cirrhosis of different aetiologies [101,102]. Intra-tumoural EMMPRIN and resultant MMP activation in tissue from HCC patients has been associated with increased HCC vascularisation, invasion, EMT, metastasis and recurrence [231,232,233,234]. With regard to specific molecular mechanisms, up-regulation of tumour EMMPRIN expression has been shown to increase tumour vascularisation, potentially via: (1) tumour and hepatic stellate cell up-regulation of growth factors, such as VEGF or IGF-II; or (2) MMP activation in hepatic stellate cells [235,236,237,238]. The resultant extensive vascularisation is a risk factor for HCC metastasis [239,240]. Further, EMMPRIN-transfected hepatocyte cell lines have been shown to up-regulate autocrine signaling of the pro-tumourigenic cytokine TGF-β and increase the rate of HCC formation when injected into nude mice compared to non-transfected controls [241]. EMMPRIN expression has also been shown to decrease the apoptosis associated with loss of cell-contact (known as anoikis) in a range of cell lines, including HCC-derived cells [242,243,244]. This process could allow survival in the circulation, enabling them to seed in distant sites and evade liver resection, ultimately resulting in early HCC recurrence.

Alteration in the Hh pathway, one of the common changes seen in HCC, has been linked with tumour-stromal signaling and subsequent tumour progression. Hh expression from the primary tumour has been found to induce dense fibrous stroma formation in primary pancreatic cancer xenografts [245] and to break down bone stroma prior to breast cancer bone metastasis [246]. In the context of HCC, the Hh pathway has been shown to up-regulate glycolysis in surrounding myofibroblasts [247]. This induces increased production of free lactate, which adjacent HCC cells may take up and feed into fatty acid metabolism pathways to derive energy. Another effect of altered Hh pathway signaling is activation of hepatic stellate cells and hepatic progenitor cells in the liver, thereby driving the fibrogenic process [248,249].

Viral proteins expressed by HBV- and HCV-infected hepatocytes have also been shown to directly alter cellular signaling to drive HCC progression. For example, overexpression of either HBV surface [154,155] or HBV X [158,159] proteins in mouse models lead to liver lesions and HCC. Furthermore, the HCV core, NS3 and NS5a proteins have been reported to directly induce a wide range of cellular effects which drive carcinogenic processes, particularly the escape of cell-cycle checkpoints [156,157,250]. However, the real impact of direct carcinogenesis by viral proteins still remains difficult to quantify and characterise as the HCC models used are usually not physiological (overexpression of HBV surface and X proteins under non-native promoters) or are unavailable (no small animal HCV model exists).

## 5. Novel Microenvironmental Factors and Their Roles in Hepatocarcinogenesis

Recent research has implicated further aspects of the microenvironment, such as diet, GIT microflora [166,251] and microvesicles derived from cell subpopulations within the liver, in driving HCC initiation and progression. Though the exact causal mechanisms are yet to be established for these factors, these are areas of HCC research that we expect to be studied in greater detail in coming years.

### 5.1. Nutrition and Diet

In addition to the established promotion of HCC by dietary AFB1, other foods and nutrients may contribute to hepatocarcinogenesis, as the liver acts as both the initial solid organ seen by nutrients absorbed by the digestive tract and a storage site for glycogen and fats. Mouse and human studies have shown that dietary iron overload may directly induce hepatocarcinogenesis [252,253], which is consistent with the HCC risk that hereditary haemochromatosis confers to patients. High fat diet and associated metabolic diseases of obesity and Type II Diabetes Mellitus (T2D) are strongly linked to non-alcoholic liver disease (NAFLD) and non-alcoholic steatohepatitis (NASH), which have been shown to be associated with fibrosis, cirrhosis and HCC in epidemiological studies [254,255,256]. This link of HCC with metabolic diseases has been illustrated with DEN-induced HCC models in mice using both genetically-obese (leptin-deficient) mice and wild-type mice fed a high-fat diet [257,258]. In both models, increased rates and size of HCC were observed. Further, obesity has been shown to be associated with earlier HCC recurrence in patients treated with liver transplantation [259]. HCC risk appears to be affected not only by the level of caloric intake, but also the composition of diet. Large population-based studies have associated reduced HCC rates with increased adherence to the “Mediterranean diet”, to increased consumption of fish, eggs, dairy, and fruits, and increased caffeine intake [260,261,262,263,264]. Together these studies suggest that diet and physical exercise to lower body mass index may protect against HCC, though we have found no reports directly testing this hypothesis.

Detailed studies investigating the specific mechanisms behind these dietary associations with HCC have been sparse to date. A potential contributor to the unclear role of dietary factors in HCC is their complicated interrelation with other known drivers of HCC [265]. For example, a synergistic increase in serum liver transaminases (a marker for liver damage) was seen in mice fed both dietary iron and ethanol [266]. Further, there is a complex relationship between hepatic steatosis, HCV (described earlier as a strong causative factor for hepatocarcinogenesis) and HCC. Patients with HCV have significantly higher rates of hepatic steatosis and fibrosis severity, compared to HBV-positive patients and healthy controls [267]. Further, HCV-positive patients with concurrent T2D were demonstrated to have a 2–3 fold increased risk of developing HCC compared to those patients without T2D [268]. This clearly indicates T2D as a risk factor for HCC; however a multivariate analysis that took other known microenvironment drivers of HCC into account found only a weak association of dietary factors to HCC progression [262,269,270]. Thus, the specific molecular roles of dietary factors in HCC development are poorly understood and are likely to be the subject to future investigations, especially with the increased worldwide incidence of metabolic diseases.

### 5.2. GIT Microflora

A high-fat diet is also associated with alterations of the resident microflora in the gastrointestinal tract [165,271,272]. It is possible that altered GIT microflora can drive HCC progression via the alteration of hepatic inflammation either by increasing the permeability of the GIT mucosa and allowing inflammatory products, such as bacterial lipopolysaccharide (LPS) or deoxycholic acid (DCA), to enter the portal circulation [165,273,274]. The promotional effect of altered gut flora on development of 7,12-dimethylbenz(a)anthracene-induced HCC in obese mice has been shown to be dependent on DCA and its alteration of hepatic stellate cell gene expression [165]. This HCC-promoting effect was not via a profibrotic hepatic stellate cell response but due to a senescence secretory response, mediated by interleukin-1β. In other studies, the growth of specific species in GIT microflora, in particular *Helicobacter* species, have been shown to promote HCC initiation and progression in mouse models of chemical and viral carcinogenesis through chronic inflammation [166,251]. Zhang *et al.* showed that alteration of gut homeostasis with penicillin significantly increased serum LPS levels, circulating pro-inflammatory cytokine interleukin-6, and both cirrhotic and HCC progression in a DEN-induced HCC rat model [166]. Similar changes were seen when the rats were treated with oral dextran sulfate sodium to increase gut permeability. Further, in this study, gut permeability could be normalised (with concomitant decrease in HCC progression) by the administration of probiotics, suggesting that clinical intervention in human patients may be possible. 

Limited human studies have reported an association between altered GIT microflora, particularly the appearance of *Helicobacter* species, with the progression of HCC and cirrhosis [275,276,277,278]. Indeed, *Helicobacter* bacteria have been found not only in the GIT, but also in the liver itself during HCC formation, though it is not clear whether this drives HCC progression or is an artefact of late-stage liver disease [275,276,277,278]. There have also been studies showing increased inflammatory bacterial metabolites LPS and DCA in patients with liver cirrhosis and HCC [165,166]. However, follow-up studies are required to determine specific causal relationships between HCC and GIT microflora and whether clinical interventions to alter GIT microflora can improve patient outcomes [279].

### 5.3. Microvesicles (Microparticles and Exosomes)

Microvesicles, including microparticles (formed by cell membrane shedding) and exosomes (formed by release of intracellular multivesicular bodies), are small, enveloped blebs that are secreted by many organs [280]. Microvesicles as carriers of cellular signals have been implicated in hepatocarcinogenesis, though the specific mechanism(s) involved are still unknown. A growing body of literature already describes the myriad of roles of microvesicles and their biological functions particularly in liver disease, ranging from systemic or intercellular communications to eliciting immunological responses [171,172].

In the context of cancer, tumour-derived microvesicles may have both anti-tumourigenic and pro-tumourigenic roles [281]. Exosomes released by cancer cells have been observed to contain tumour antigens, leading to the hypothesis that anti-cancer vaccines could be developed from these tumour-derived exosomes. Several phase I clinical trials have used tumour-derived exosomes or exosome-pulsed dendritic cells as cancer vaccines [282,283]. In addition to their immunostimulatory effects, these exosomes may also be involved in tumour cell apoptosis via decreased B-cell lymphoma 2 protein (Bcl-2) and increased Bcl-2-associated X protein (Bax) expression [284]. Specifically in HCC, heat-shock-protein (HSP)-bearing exosomes are released from human HCC cell lines upon stimulation with chemotherapeutic agents, and these exosomes can elicit natural killer cell anti-tumour responses [285].

The pro-tumourigenic functions of microvesicles include immunosuppressive effects via T_reg_ cells and inhibition of differentiation of bone marrow dendritic cells [169,170]. Further, exosomes can facilitate tumour-invasion and metastasis by up-regulation of tetraspanins (a class of phospholipid membrane molecules known to promote angiogenesis) [286,287], and can transport mRNA, microRNAs and proteins essential for tumour survival and growth [173,174]. Interestingly, microvesicles from tumour cells [167] (including HCC cell lines [288]) and circulating T-cells [168] are known to contain EMMPRIN and stimulate breakdown of ECM by hepatic stellate cells and fibroblasts.

In HCC, the effects of exosome-transported mRNAs and miRNAs have not been completely elucidated. Microvesicles released from human liver stem cells have been shown to inhibit HCC growth in SCID mice [289]. This effect was reportedly due to delivery of miRNAs (miR451, miR223, miR24, miR31, miR214 and miR122), which conferred direct anti-tumour activity via down-regulation of MDR1, MIF, RAB14 and E2F-2. TGF-β activated kinase-1 (TAK1) has also been identified as a potential pathway modified by miRNA carried by microvesicles [290], as loss of TAK1 has been implicated in hepatocarcinogenesis and is a plausible target for intercellular modulation. We are unaware of any studies to date showing that HCC-specific microvesicles in the liver microenvironment alter the phenotype of surrounding non-tumour hepatocytes, though this is a potential, even likely, route of cell-cell signaling.

It is increasingly apparent that microvesicles are intricately linked to various factors in the tumour microenvironment that may promote local spread, intrahepatic metastases, and possibly multifocal growth of HCC. Although the molecular pathophysiological mechanisms are not entirely understood at present, the microvesicular cargo and membrane proteins may be pivotal in intercellular communication and modification of tumour behaviour.

## 6. Targeting the Microenvironment to Prevent and Treat HCC

By characterising the effect of microenvironmental factors on HCC initiation and development further, we may be able to target HCC in multiple ways to synergistically decrease development and spread of the tumour. Indeed, existing measures against microenvironmental factors have been used to prevent and treat HCC.

The most successful HCC prevention measure to date has been vaccination against HBV. Vaccination in highly endemic areas, such as Taiwan, has led to a large drop in HCC cases [20,291,292]. Since HBV/HCV infection is a risk factor for HCC recurrence after resection [293,294], several groups have studied the rate of HCC recurrence after antiviral treatment [295]. The general consensus is that antiviral treatment significantly decreases HBV/HCV-associated HCC recurrence. However, the specific anti-viral therapy agent(s), dose, timing, and routes all require further optimisation [296,297,298]. In HCV infection, treating active virus infection and achieving a sustained virological response (SVR) is associated with a 75% reduction in HCC risk despite the persistence of cirrhosis. Therefore, the grade of hepatitis activity is a risk factor for HCC recurrence and the main mechanism of antiviral treatment may be freeing the liver microenvironment from drivers of chronic inflammation, and thereby preventing recurrent HCC. Cofactors in HCC progression, including obesity and diabetes, alcohol, iron overload and viral co-infections, are clearly important and may increase risk of HCC through further promotion of inflammation as well as having other unrecognised effects on carcinogenesis. A highly relevant and important example is the increased risk of HCC with increasing HBV DNA levels [293,294,295,296,297,298,299,300], which is associated with greater inflammation. Other factors, such as the serum level of HBV surface antigen, genotype of the virus and expression of HBV e-antigen, all add to the risk for the occurrence of HCC [301,302,303].

HCC progression can potentially be limited by normalising the liver microenvironment using other strategies. For example, treatment with angiogenic agents may normalise existing aberrant vascularisation in the tumour and therefore to limit hypoxic regions [218]. Reducing the number of hypoxic cells may limit tumour progression by improving access of chemotherapies and by decreasing the expression of hypoxia-induced genes in tumour subpopulations that promote metastasis [304]. Several pro-drugs that are converted into cytotoxic agents by low intracellular oxygen levels have been designed and tested up to phase II and phase III trials, with varying levels of success [305]. Also, in addition to being an inhibitor of HCC growth, Sorafenib acts as an anti-fibrotic agent in early stages of fibrosis in mouse models [306]. These agents may limit the formation of hypoxic regions and thus block some pro-oncogenic pathways in the liver. Furthermore, altering the expression or activity of molecules known to be associated with cancer progression may have utility. For example, blocking EMMPRIN signaling using antibodies has been shown to reduce invasion in cell lines, reduce tumour size in experimental HCC mouse models and decrease fibrosis in CCl_4_-treated mice [228,307]. Further, radiolabelled anti-EMMPRIN antibodies have been shown to be well-tolerated and decrease tumour recurrence in HCC patients, however the mechanism, whether due to blocking EMMPRIN function, enhancing delivery of radio-therapeutics or both, is unclear [308,309].

Attempts have been made to stimulate the poor tumour-specific cytotoxic immune responses present in HCC patients [310,311]. Some studies have used either HCC lysates or specific HCC antigens, such as AFP, as vaccines or as antigens to be loaded onto dendritic cells *ex vivo* to be reintroduced into the host. While successful in mice [312], early trials conducted on patients with late-stage HCC have shown that tumour-specific immune responses can be raised with these approaches, but with little improvement in clinical outcomes [313,314]. Also, post-resection HCC recurrence was measured in in HCC patients after isolation of circulating lymphocytes and reperfusion following their activation with interleukin-2 and anti-CD3 antibodies [315]. The treated patients had extended recurrence-free time intervals and a reduced risk of recurrence, but overall survival was not significantly different from non-treated controls. Local tolerogenic factors in the tumour may be contributors to the poor clinical outcomes seen in these trials and may have to be modulated to optimise response. For example, Greten *et al.* depleted T_reg_ in HCC patients by treatment with cyclophosphamide, resulting in an increase in HCC-specific T-cell responses [316].

## 7. Conclusions

In summary, HCC arises not simply due to a hepatocyte containing sufficient genetic mutations, but also develops due to its context in the liver microenvironment. A multitude of inter-related factors in the liver microenvironment affect the progression of HCC, with some these mechanisms still to be fully described. Characterisation of the mechanisms of these novel factors has driven the pursuit toward new therapies that treat not only the tumour itself, but also the liver microenvironment to prevent the recurrence and treatment resistance associated with HCC.
